# Experimental immunization of mice with a recombinant bovine enterovirus vaccine expressing BVDV E0 protein elicits a long-lasting serologic response

**DOI:** 10.1186/s12985-020-01338-6

**Published:** 2020-07-01

**Authors:** Xiao Ren, Shan Zhang, Xintao Gao, Xiaoyu Guo, Ting Xin, Hongfei Zhu, Hong Jia, Shaohua Hou

**Affiliations:** 1 Institute of Animal Sciences, Chinese Academy of Agricultural Sciences, No. 2, Yuan Ming Yuan West Road Haidian District, Beijing, 100193 China; 2 Biotechnology Research Institute, Chinese Academy of Agricultural Sciences, No. 2, Yuan Ming Yuan West Road Haidian District, Beijing, 100193 China

**Keywords:** Bovine enterovirus, Bovine viral diarrhea virus, E0 gene, 2C/3A junction, Viral vector

## Abstract

**Background:**

Bovine viral diarrhea virus (BVDV) is a cause of substantial economic loss to the cattle industry worldwide, and there are currently no effective treatment or preventive measures. Bovine enterovirus (BEV) has a broad host range with low virulence and is a good candidate as a viral vaccine vector. In this study, we explored new insertion sites for the expression of exogenous genes in BEV, and developed a recombinant infectious cDNA clone for BEV BJ101 strain expressing BVDV E0 protein.

**Methods:**

A recognition site for the viral proteinase 3C^pro^ was inserted in the GpBSK-BEV plasmid at the 2C/3A junction by overlapping PCR. Subsequently, the optimized full-length BVDV E0 gene was inserted to obtain the recombinant infectious plasmid GpBSK-BEV-E0. The rescued recombinant virus was obtained by transfection with linearized plasmid. Expression of BVDV E0 in the recombinant virus was confirmed by PCR, western blotting, and immunofluorescence analysis, and the genetic stability was tested in MDBK cells over 10 passages. We further tested the ability of the recombinant virus to induce an antibody response in mice infected with BVDV and immunized them with the recombinant virus and parental strain.

**Results:**

The rescued recombinant virus rBEV-E0 was identified and confirmed by western blot and indirect immunofluorescence. The sequencing results showed that the recombinant virus remained stable for 10 passages without genetic changes. There was also no significant difference in growth dynamics and plaque morphology between the recombinant virus and parental virus. Mice infected with both recombinant and parental viruses produced antibodies against BEV VP1, while the recombinant virus also induced antibodies against BVDV E0.

**Conclusion:**

A new insertion site in the BEV vector can be used for the prevention and control of both BEV and BVDV, providing a useful tool for future research on the development of viral vector vaccines.

## Background

Bovine enterovirus (BEV) was first reported in 1957 and has been prevalent in major cattle breeding countries and regions worldwide, including the United States, Canada, the United Kingdom, Germany, Poland, Pakistan, and China [1–3]. Infection with BEV typically manifests as subclinical symptoms, but can cause diarrhea and respiratory diseases in certain cases [4]. BEV uses a wide range of animals as hosts, including bovine [5], moose [6], sheep [7], opossums [8], and dolphins [9]. In certain cases, human blood samples have also tested positive for the virus [10]. According to the latest classification of viruses, bovine enterovirus is divided into groups EV-E and EV-F, with EV-E four subtypes (EV-E1–E4) and EV-F divided into six subtypes (EV-F1–F6) [11]. BEV is a member of the genus *Enterovirus* in the family *Picornavirus.* The virion is spherical, icosahedral, non-encapsulated, and has a diameter of 25–30 nm. The viral genome is a non-segmented single-stranded positive-stranded RNA with a total length of about 7.5 kb. It can directly translate a polyprotein as mRNA and undergoes a series of degradation steps to produce four structural egg proteins (VP1–VP4) and seven non-structural proteins (2A^pro^, 2B, 2C, 3A, 3B, 3C^pro^, and 3D), among which the viral protease 3C^pro^ recognizes and cleaves a characteristic amino acid sequence (ALPQG) within exposed and flexible structural domains [12, 13].

BEV is generally considered to be non-virulent or of low virulence, not highly pathogenic, resistant to acidic environments, and can infect animals through the intestinal tract, making it a good candidate for a vaccine vector. Despite substantial progress in the development of recombinant and chimeric human enteroviruses such as poliovirus and EV-71 virus [14], relatively little research has been done on BEV in this regard. Chang et al. [15] inserted the foot-and-mouth disease virus (FMDV) type O-conserved neutralizing epitope 8E8 into the VP1 B-C or D-E loops of BEV (BHM26 strain). Chu et al. [16] inserted the main antigen neutralization epitope (residues 141–160) of the FMDV (vaccine strain O1/Manisa/Turkey/69) VP1 gene into the junction of VP1/2A of BEV (LC-R4 strain). Liu et al. [17] constructed a recombinant infectious BEV clone by insertion of the epitope of influenza virus hemagglutinin (HA) into the 3A or VP1 gene of BEV (HY12 strain), respectively. These studies indicated that the biological characteristics of recombinant BEV are similar to those of the parental virus, and experimental infection animal models could produce immune responses to the exogenous genes. However, these studies only explored some of the circulating strains and potential insertion sites, and many other strains and other highly effective potential insertion sites remain to be investigated.

Bovine viral diarrhea virus (BVDV), a single-stranded positive-strand RNA virus belonging to the family *Flaviviridae* and the genius *Pestivirus,* is the cause of bovine viral diarrhea (BVD). BVD is a complex disease with various clinical manifestations and is considered one of the main threats to the cattle industry worldwide. BVDV not only infects cattle but also infects sheep, goats, pigs, deer, and other ruminants with a wide host range [18]. Since there is currently no specific treatment for BVD, it is particularly important to find new measures to prevent its occurrence and transmission. The BVDV E0 gene is highly conserved in the BVDV genome and has a neutralization epitope [19], which can generate the production of a neutralization antibody to neutralize BVDV. Therefore, we explored the possibility of BVDV E0 as a candidate antigen for the genetic engineering of a subunit vaccine using BEV.

In this study, we used the BEV BJ101 strain as a viral vector to express exogenous genes by inserting the BVDV E0 gene between the genes encoding the non-structural proteins 2C and 3A. We compared the replication, infection, and biological characteristics of the rescued recombinant virus after insertion to those of the parental virus, and evaluated the expression of E0 protein and its ability to induce an immune response in vivo.

## Methods

### RNA extraction and cDNA synthesis

Viral RNA was extracted from Madin-Darby bovine kidney (MDBK) cells (provided by Hebei Agricultural University) infected with the BEV BJ101 strain (maintained in our laboratory) using an RNA extraction kit (AXYGEN, Hangzhou, China). The first-strand cDNA of the virus was synthesized with the PrimeScript™ II 1st Strand cDNA Synthesis kit (Takara, Beijing, China).

### Optimization and synthesis of BVDV E0

Mammalian codon optimization was carried out on the gene sequence of BVDV E0 (GenBank: EU709763.1) and the optimized sequence is shown in Additional File 1. A *Not*I enzyme cleavage site was added to the 5′ end and an *Xba*I enzyme cleavage site was added to the 3′ end. The whole gene sequence was connected to the pUC-57 plasmid vector and named pUC57-E0.

### Construction of infectious cDNA clones of the recombinant virus

**Fig. 1:**
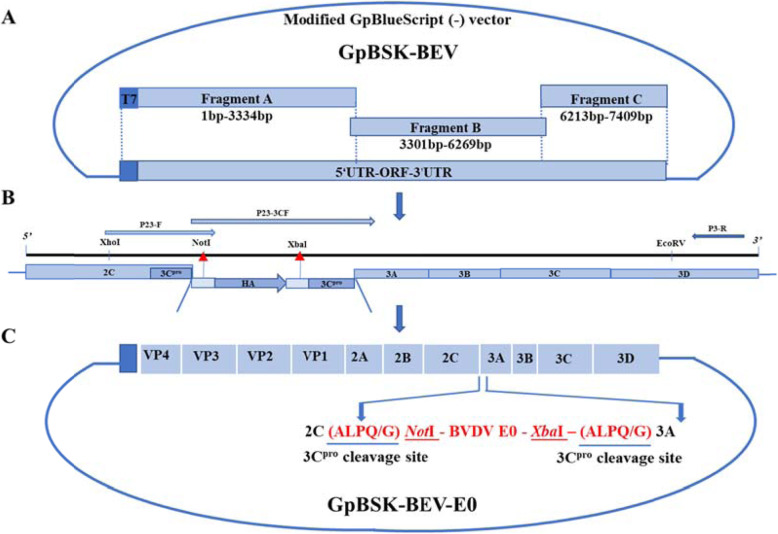
Construction and strategy of the GpBSK-BEV and GpBSK-BEV-E0 plasmids. (**a**) Construction of the GpBSK-BEV plasmid; (**b**) The insertion of *Not*I and *Xba*I double restriction sites and a recognition site for 3C^pro^ into the 2C/3A junction in the GpBSK-BEV plasmid; (**c**) Construction of the GpBSK-BEV-E0 plasmid, the 3C^pro^ cleavage site (ALPQG) and *Not*I and *Xba*I enzyme double restriction sites are underlined

In accordance with the strategy schematically outlined in Fig. 1, a full-length BEV BJ101 infectious cDNA clone harboring the exogenous gene BVDV E0 was generated by inserting the optimized nucleotide sequence of BVDV E0 at the 2C/3A junction of the virus.

The synthesized cDNA was used as a template for subsequent polymerase chain reaction (PCR) amplification. The genome sequence of BEV BJ101strain (GenBank: MG650158.1) was divided into three fragments, fragment A, B and C, containing nucleotides 1–3334, 3301–6269, and 6213–7409, respectively, for amplification. Three pairs of specific primers (A-F/R, B-F/R, C-F/R) were designed to amplify the three fragments, and cloned into the modified GpBlueScript (−) vector (stored in our laboratory; see Additional File 2) by homologous recombination to generate the full-length cDNA clone of GpBSK-BEV (Table 1).

**Table 1:** Primers for the construction of full-length cDNA clone of BEV

Primer name	Primer sequence (5′ to 3′)	Product size (bp)
BEV-AF	GTAAAACGACGGCCAGTGAATTGGTCGACTAATACGACTCACTATAGGGTTAAAACAGCCTGGGGGTTG	3383
BEV-AR	GGGCCCAATGTGGTCAGGGAGGATCGATCGGTGCAGATCTTATCGGTG	
BEV-BF	GCACCGATCGATCCTCCCTGACCACATTGGGCCCATTTGGAC	2969
BEV-BR	GCCTTCAGTACCATAAATGGCATCCTCCATGGATAT**C**GGCCTAGG	
BEV-CF	CTAAATATTGATCCTAGGCCGATATCCATGGAGGATGCC	1249
BEV-CR	CTCGAAATTAACCCTCACTAAAGGGAACGAATTCTTTTTTTTTTTTTTTTTTACACCCCATCCGGTG	

The *Not*I and *Xba*I double restriction sites and a recognition site for 3C^pro^ were inserted at the 2C/3A junction in the GpBSK-BEV plasmid by overlapping PCR (Table 2). In brief, the GpBSK-BEV plasmid was used as the first template, P23-3CF and P3-R were the primers, and PCR was performed in accordance with PrimeSTAR® HS (Premix) specifications (Takara, Beijing, China). The amplified product served as the subsequent template using the primers P23-F and P3-R for the second PCR. The obtained product and GpBSK-BEV plasmid were then subjected to double enzyme digestion using the *Xho*I and *EcoR*V restriction enzymes (Takara, Beijing, China) present in the original plasmid. An HA tag was added between the *Not*I and *Xba*I restriction sites to prevent the proximity of the two enzyme cleavage sites from affecting the efficiency of double enzyme cleavage. Finally, the GpBSK-BEV plasmid containing *Not*I and *Xba*I enzyme cleavage sites was obtained through a T4 ligase (Takara, Beijing, China) connection.

**Table 2:** Primers for overlapping PCR

Primer name	Primer sequence (5′ to 3′)	Product size (bp)
P23-F	GCAATAGGCGTTATAACATTGGGAACGTCCTCGAGGCACTCTTCCAGGGCGGCCGCTACCCATACGAC	1317
P23-3CF	G***GCGGCCGC***TACCCATACGACGTCCCAGACTACGCT***TCTAGA***GCACTCTTCCAGGGCCCAGTTTGCTACAAACCCCTC	1270
P3-R	CCAGGGCCTCCAAGCCTTCAGTAC	

**Note:***Not*I (GCGGCCGC) and *Xba*I (TCTAGA) enzyme double restriction sites are italic, bold and underlined

The pUC57-E0 and GpBSK-BEV plasmids were digested with *Not*I and *Xba*I enzymes (Takara, Beijing, China), respectively. Subsequently, T4 ligase was used for overnight ligation at 16 °C, and the product was transferred into the competent cells of *Escherichia coli* JM109 (Sangon, Shanghai, China). Positive clones were sent to Genewiz (Suzhou, China) for sequencing, and the correct plasmid was named GpBSK-BEV-E0.

### Linearization of the recombinant plasmid

The recombinant plasmid GpBSK-BEV-E0 was linearized by the *Eco*RI restriction enzyme (Takara, Beijing, China). The enzyme digestion system comprised 10 μg recombinant plasmid, 40 μL of 5× CutSmart buffer, 8 μL *Eco*R I-HF, and RNase Free ddH_2_O up to 200 μL. Enzyme digestion was performed at 37 °C for 3 h, and the restriction enzyme was inactivated at 65 °C for 20 min. Linear plasmids were recovered from the agarose gel and purified by ethanol precipitation.

### Transfection and rescue of the recombinant virus

Linearized GpBSK-BEV-E0 was transfected in BSR-T7 cells (provided by Hebei Agricultural University) using a transfection kit (Polyplus, NA, USA) according to the manufacturer’s instructions. In brief, BSR-T7 cells were inoculated into a six-well plate until covering 80% of the bottom of the wells, and then washed with phosphate buffered saline (PBS). Two milliliters of Dulbecco’s modified Eagle’s medium (DMEM; Life Technologies, CA, USA) containing 2% fetal bovine serum (FBS; Sigma-Aldrich, St. Louis, MO, USA) was added to each well, and 200 μL of jetPRIME buffer and 2 μg DNA were added to the sterilized 1.5-mL tube and mixed gently. Subsequently, another 4 μL of jetPRIME was added, mixed gently, centrifuged at low speed for 10 s, and left to react at room temperature for 10 min. The mixture was then added to a six-well plate and cultured in the cell incubator at 37 °C and 5% CO_2_. Viruses were observed at 12-h intervals and harvested until cytopathic effects (CPE) were visible.

### Preliminary identification of the recombinant virus

Specific detection primers of BEV (BEV-F/R) and BVDV E0 (E0-F/R) were designed (Table 3) and applied for PCR identification of the recombinant virus with 2 × Taq Master Mix (Dye) kit (Vazyme, Naijing, China). The identified recombinant plasmid was sent to Genewiz (Suzhou, China) for sequencing.

**Table 3:** Specific primers for detection

Primer name	Primer sequence (5′ to 3′)	Product size (bp)
BEV-F	TTAAAACAGCCTGGGGGTTGTACC	696
BEV-R	TTTACACCCCATCCGGTGGGTG	
E0-F	AGATGGAATGAGATACAGCTTGG	901
E0-R	GGAACGACAGTACTCTCGGA	

### Detection of the recombinant virus by western blot

The rescued recombinant virus rBEV-E0 was also detected by western blot according to standard procedures. In brief, MDBK cells were infected with the 10th generation recombinant virus and parental virus, and incubated for 12 h. Viral samples were loaded on a 12% polyacrylamide gel, transferred to nitrocellulose membranes, and then blocked with 5% skim milk in Tris-buffered saline with Tween 20 for 2 h at 37 °C. The membranes were then incubated with anti-BJ101-VP1 monoclonal antibody (mAb) 4B2 and anti-BVDV E0 polyclonal antibody (PcAb, 1:3000; both prepared in our laboratory) at 37 °C for 1 h followed by incubation with horseradish peroxidase (HRP)-conjugated goat anti-mouse antibody (1:4000; Sigma-Aldrich). Detection was performed using an enhanced chemiluminescence kit (Beyotime, Shanghai, China).

### Detection of the recombinant marker viruses by indirect immunofluorescence analysis (IFA)

The rescued recombinant virus rBEV-E0 and parental virus were transfected in MDBK cells in 96-well plates. After 24 h, the cells were fixed with ice-cold anhydrous ethanol at 4 °C for 30 min, followed by the addition of 0.3% Triton 100 for 15 min. Anti-BEV VP1 mAb and anti-BVDV E0 PcAb (1:1000) were added and the cells were further incubated at 37 °C for 1 h, followed by incubation with fluorescein isothiocyanate-conjugated goat anti-mouse secondary antibody (1:500; Sigma-Aldrich) in the dark. The results were observed under a fluorescence microscope.

### Plaque morphology of the recombinant viruses

MDBK cells in the six-well plate were infected with the 10th generation recombinant virus and parental virus by 10-fold gradient dilution at 37 °C for 2 h. After removing the unabsorbed virus, the cells were overlaid with 2 mL of 1.5% low-melting agarose containing 2% FBS in DMEM and cultured at 37 °C in 5% CO_2_ for 96 h. The plaques of moderate size were selected by staining with 0.1 g/L neutral red solution.

### Virus propagation dynamics

The growth curves of the recombinant virus and parental virus in MDBK cells were compared. In brief, the MDBK cells in 96-well plates were infected at a multiplicity of infection of 1 with the 10th-generation rescued virus or parental virus and incubated at 37 °C in 5% CO_2_ for 1 h. After adsorption, the cells were washed with PBS and added to DMEM containing 2% FBS. Infected cells were harvested at 3, 6, 12, 24, 36, and 48 h after infection, and then frozen and thawed three times. The 50% tissue culture infective dose (TCID_50_) of the virus was calculated according to the Reed-Muench method [20] and each sample was run in triplicate for determination.

### Determination of the stability of the recombinant virus

The stability of the rescued recombinant virus was determined by PCR and sequencing. RNA was extracted from MDBK cells infected with the 3rd, 5th, 7th, and 10th generation recombinant viruses. RNA was then reverse-transcribed into cDNA and used as a template for PCR detection with primers (Table 3). The obtained PCR amplification fragment was sequenced to further identify the BVDV E0 gene in the rescued virus.

### Immunogenicity of the rescued recombinant virus

Twenty-four BALB/c female mice (6–8 weeks old; Charles River, Beijing, China) were randomly divided into four groups of six mice each. The test group was intraperitoneally injected with 10^8^ TCID_50_ of rBEV-E0 and the positive control groups were respectively immunized with the same dose of BVDV and BEV. The final group was a blank control group immunized with PBS. The activity of the mice was observed every day, and blood samples were collected before immunization (D0) and at 7-day intervals after immunization.

The blood samples were separated into serum, and indirect enzyme-linked immunoassay (ELISA) was used to detect anti-BEV VP1 and anti-BVDV E0 antibodies in the immunized mice. The 96-well ELISA plates were coated with VP1 and E0 proteins at 100 ng/mL each and incubated overnight at 4 °C. The wells were blocked with PBS and Tween containing 1% bovine serum albumin for 2 h at 37 °C. Serum samples serially diluted two-fold starting at a dilution of 1:500 were added and incubated at 37 °C for 1 h, followed by the addition of HRP-conjugated goat anti-mouse secondary antibody. Finally, 3,3′,5,5′-Tetramethylbenzidine (TMB) was added for color development, and the reaction was terminated by the addition of 2 mol/L H_2_SO_4_. The optical density at 450 nm (OD_450_) cutoff values were determined for analysis.

### Statistical analysis

Statistical analysis was performed using GraphPad Prism 6 software (GraphPad Software, CA, USA). Data were analyzed using two-way analysis of variance (ANOVA). Data are shown graphically as the geometric mean of the fold change plus the standard error of the mean. A *P* value of < 0.05 was considered significant.

### Ethical approval

All animal experimental procedures were approved by the Animal Care and Use Committee of the Institute of Animal Sciences of Chinese Academy of Agricultural Sciences.

## Results

### Insertion of BVDV E0 in the BEV genome

After generating recombinant infectious BEV-E0 cDNA clones (GpBSK-BEV-E0) following the strategy outlined in Fig. 1, the PCR and sequencing results (Table 3; sequencing results not shown) confirmed that the recombinant infectious clone contained the BVDV E0 gene at the correct position.

### Rescue of the recombinant marker viruses

**Fig. 2:**
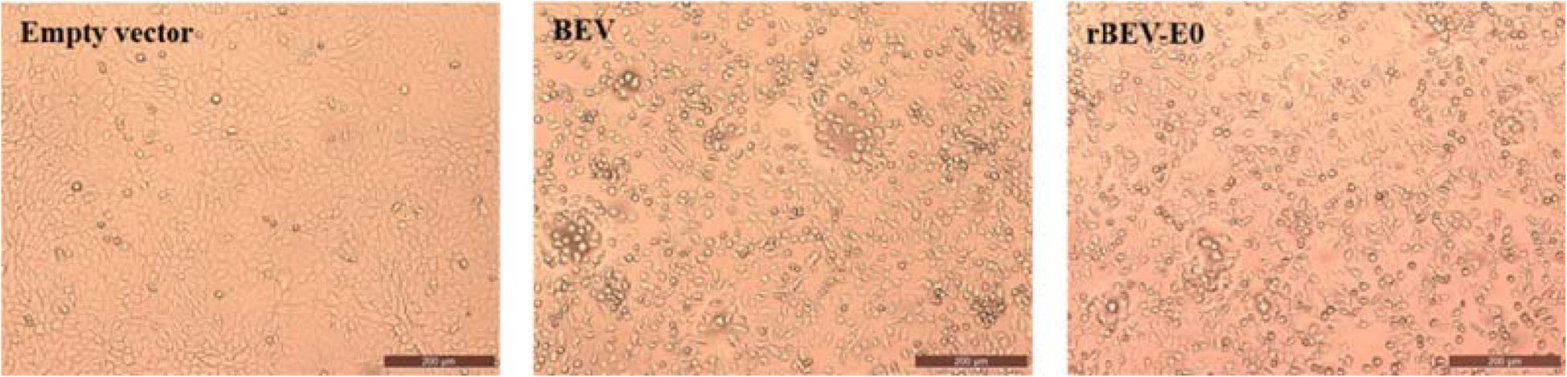
CPE after infection with rescued recombinant virus. CPE was observed in MDBK cells transfected with linearized GpBSK-BEV-E0 plasmid. The parental BEV BJ101 and GpBlueScript (−) empty vector was respectively used as a positive and negative control

**Fig. 3:**
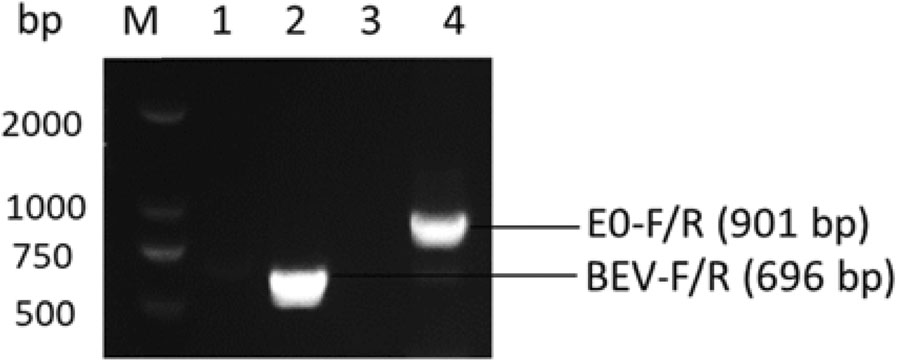
The PCR identification of rescued recombinant virus. The primer pair BEV-F/R was used in line 1 and line 2 and E0-F/R used in line 3 and line 4. ddH_2_O was used as template for negative control (line 2 and line 4)

As shown in Fig. 2, at 24–36 h after transfection of linearized GpBSK-BEV-E0, the BSR-T7 cells showed CPE (mainly manifesting as cell granular lesions, shrinking, rounding and falling off), which were similar to those observed in the cells transfected with the GpBSK-BEV plasmid; however, no CPE was observed in the cells transfected with the empty vector. To confirm that the rescued recombinant virus contained BVDV E0, the sequence across the insertion site of BVDV E0 was amplified by PCR (Fig. 3), and the sequencing results confirmed the existence of BVDV E0.

### Expression of BVDV E0 in cells infected with recombinant viruses

**Fig. 4:**
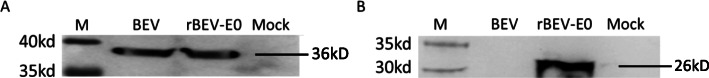
The Western Blot identification of rescued recombinant virus. Expression of BVDV E0 was confirmed with Western blot analysis. MDBK cells infected with parental BEV BJ101 and empty MDBK cells were respectively used as a positive and negative control. Anti-VP1 mAb and anti-E0 PcAb were respectively used in Fig. 4**a** and Fig. 4**b**

Western blots showed a band for VP1 in the MDCK cells infected with both the parental and recombinant viruses (Fig. 4a), whereas the uninfected cells did not produce a band. Using anti-E0 PcAb, only one band appeared in the cell samples infected with the recombinant virus, and no band was observed in the uninfected cells or in the cells infected with the parental virus (Fig. 4b).

**Fig. 5:**
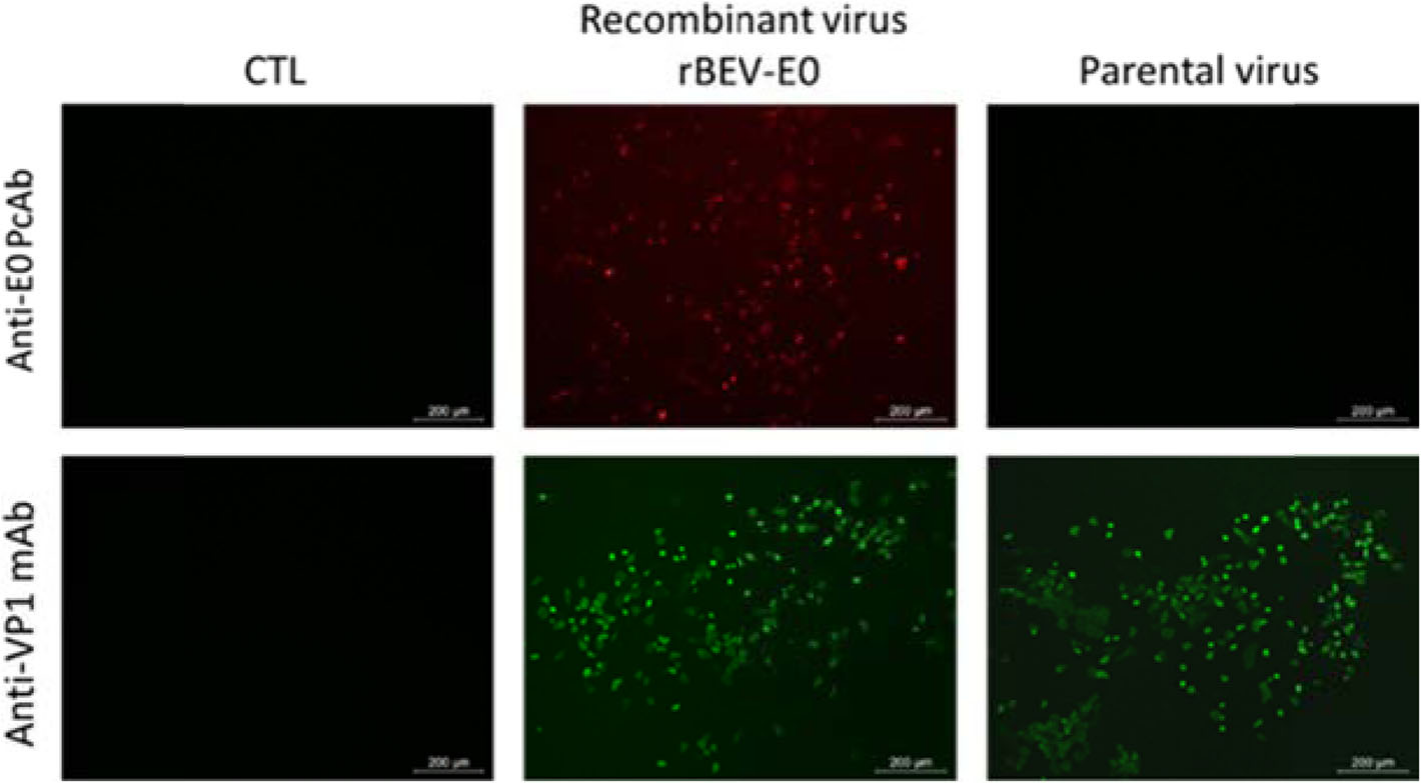
Indirect IFA detection of MDBK cells infected with rescued recombinant virus. Anti-E0 PcAb and anti-VP1 mAb were used in the experiment. Parental BEV BJ101 was used as positive control. MDBK cells infected with parental BEV BJ101 and empty MDBK cells were respectively used as a positive and negative control

Indirect IFA confirmed these results, as the samples infected with recombinant virus or parental virus all reacted with anti-VP1 mAb. The samples infected with the recombinant virus only reacted with anti-E0 PcAb, and the uninfected samples did not respond against either anti-VP1 mAb or anti-E0 PcAb (Fig. 5). These results confirmed that the inserted exogenous gene BVDV E0 was normally expressed in the cells infected with the recombinant virus.

### Plaque morphology

**Fig. 6:**
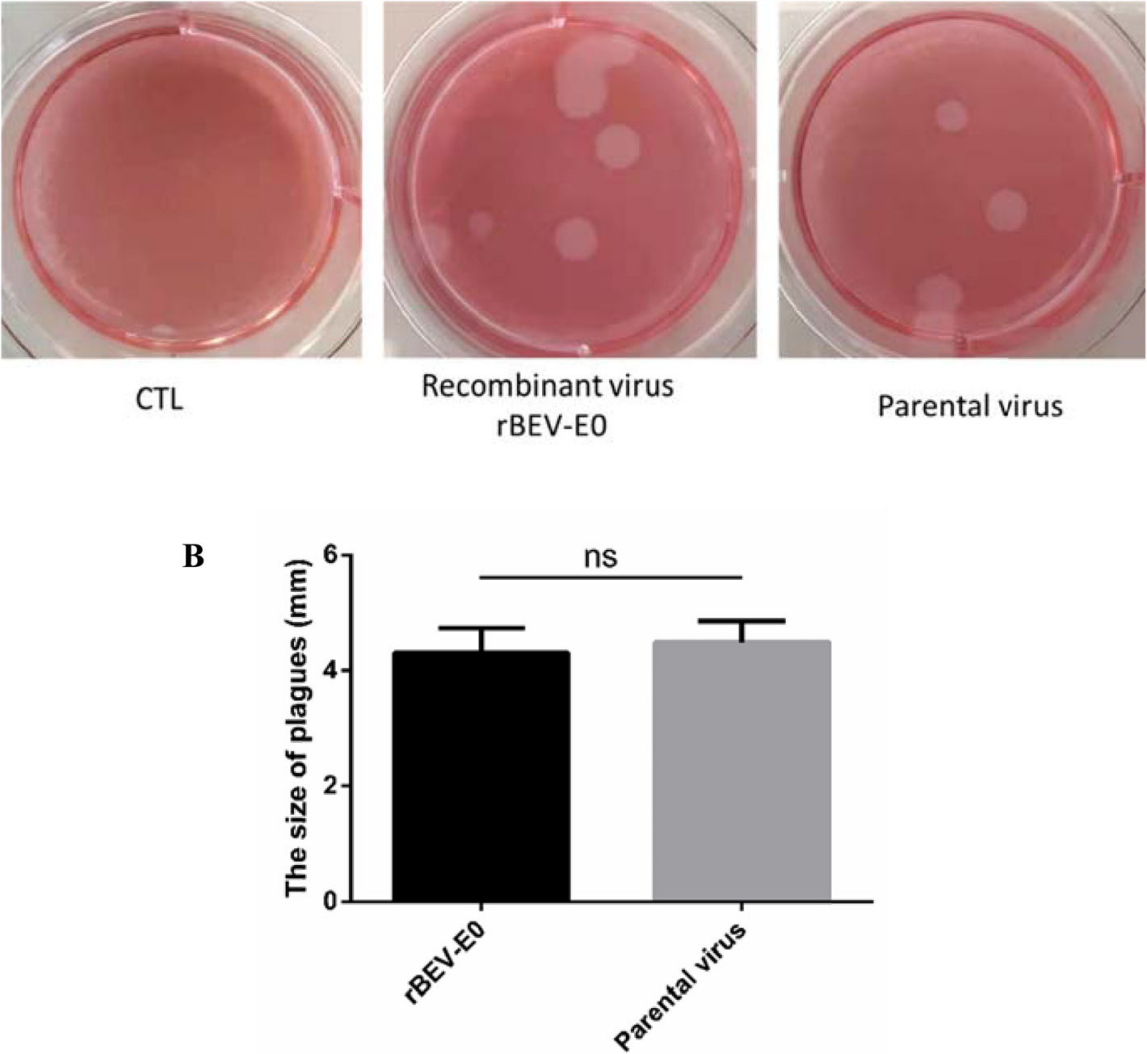
Morphology of plaques formed by rescued recombinant virus in MDBK cell monolayers. The plaque size and morphology of recombinant virus was not significantly different from that of the parental virus. The representative plaques and variance analysis were respectively displayed in Fig. 6**a** and Fig. 6**b**

After infection with the recombinant virus and parental virus for 72 h, obvious CPE was observed in MDBK cells. After staining with neutral red solution, various plaques of different sizes were visible to the naked eye, whereas no plaques appeared in the control group (Fig. 6a). The recombinant virus (Size of plagues: 4.309 ± 0.4358 mm, *N* = 7) showed a plaque morphology similar to that of the parental virus (Size of plagues: 4.495 ± 0.3649 mm, *N* = 4), and the difference is not significant (*p* > 0.05, Fig. 6b).

### Biological characteristics of the recombinant virus

**Fig. 7:**
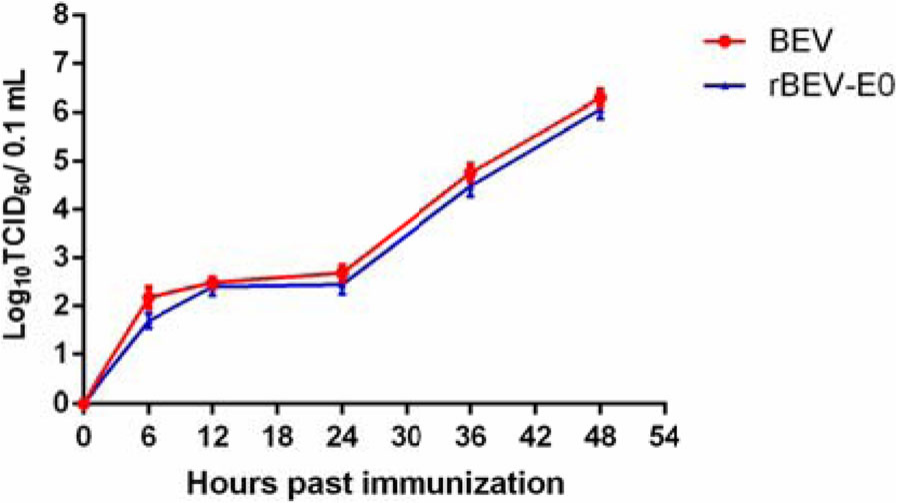
One-step growth curves of rescued recombinant virus in MDBK cells. MDBK cells were infected with rBEV-E0 recombinant viruses and parental BEV, at a MOI of 1. The data from three independent experiments are expressed as Log_10_TCID_50_ /0.1 mL. Error bars represent standard error of the mean (SEM)

As shown in Fig. 7, the growth characteristics and replication efficiency of the recombinant virus were similar to those of the parental virus, suggesting no effect of the insertion of BVDV E0 at the 2C/3A junction on the biological characteristics of the virus.

### Genetic stability of the recombinant viruses

**Fig. 8:**
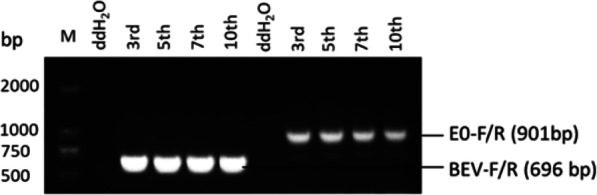
The genetic stability detection of rescued recombinant virus. PCR with the primer pairs (BEV-F/R and E0-F/R) was performed on the 3rd, 5th, 7th, and 10th generations of recombinant virus rBEV-E0. ddH_2_O was used as template for negative control

PCR performed on the 3rd, 5th, 7th, and 10th generations of recombinant viruses showed that the recombinant virus could amplify both the specific detection fragments of BEV and E0 (Fig. 8). All of the recombinant viruses retained the foreign gene BVDV E0 during the passage of MDBK cells, demonstrating the stability of the insertion of BVDV E0 at the 2C/3A junction.

### In vivo antibody response to the recombinant viruses

**Fig. 9:**
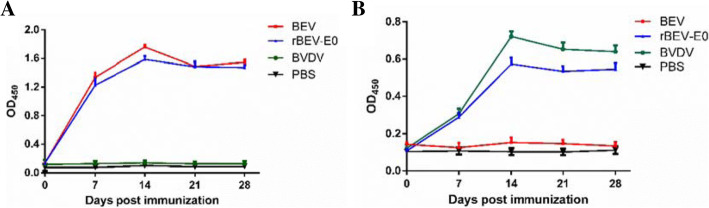
Antibodies detection in mice infected with rescued recombinant virus. Anti-BEV VP1 and anti-BVDV E0 antibodies were detected by ELISA. The sera were collected from mice inoculated with the parental or recombinant marker viruses at 0, 7, 14, 21 and 28 days. (**a**) BEV-VP1 protein-based ELISA. (**b**) BVDV-E0 protein-based ELISA. Data are presented as the mean ± SEM in the same treatment

No obvious clinical signs were observed throughout the observation period, as described in other references. As shown in Fig. 9, at D7 after intraperitoneally injection with recombinant or parental viruses and BVDV, both the rBEV-E0 group and the BEV group produced anti-VP1 antibody. The BEV group reached a high antibody level during D14–D21, and the rBEV-E0 group did not reach a corresponding level until D21. In addition, the antibody level in the rBEV-E0 group was lower than that in the BVDV group at the same detection time point, but the difference was not statistically significant. These results showed that mice immunized with rBEV-E0 produced an effective immune response.

## Discussion

Enterovirus is a pathogen that can cause diseases of different degrees, and reverse genetic technology is now widely used to investigate the enterovirus structure and protein function toward the development of viral vector vaccines. Viral vectors are commonly used to deliver genetic material into cells; however, the selection of appropriate viral vectors has long been a major constraint in the development of gene therapy and vector vaccines. An additional challenge is the difficulty in effectively stimulating mucosal immunity using chemically inactivated vaccines. However, BEV, an intestinal pathogen that usually infects animals through the intestines or respiratory tract, is an ideal choice to overcome this problem. BEV is widely distributed in animals, and results in only mild or no clinical symptoms [21]. In addition, BEV has an oncolytic property and can be used as drug therapy [22]. Therefore, BEV is a good candidate to develop a live virus vector for expressing foreign antigens.

In previous study [23], we had obtained the infectious clone of BJ101 strain and successfully rescued the virus. By comparison tests, it was shown that the rescued virus, rBEV had similarity with the parent strain in host tropism, plaque morphology, biological characteristics and genetic stability. This is a preliminary preparation for this research.

The BEV genome contains a large open reading frame encoding four structural proteins and seven non-structural proteins. Previous studies explored various potential insertion sites for exogenous genes in BEV vectors, including the junction of the VP1 and 2A genes within the 2A, 2C, 3A, and VP1 genes, and in the VP1 B-C or D-E loop [15–17]. Early studies showed no adverse effects on viral biology or replication after insertion of exogenous epitopes at these sites. In this study, we used a reverse genetics platform to explore the feasibility of using BEV as a viral vector to express the BVDV E0 gene, and found a new suitable insertion site (2C/3A). The 2C protein is highly conserved among enteroviruses, which exhibits ATPase activity and can bind to RNA and the membrane, and has been associated with the virulence of viruses [24]. The 3A protein is derived from a 3AB precursor protein and is found as an isomeric dimer. In the process of viral infection, 3A protein prevents the formation of host proteins and connection with the membrane, and deletion and mutation of a hydrophobic area at the C-terminal of 3A affects replication of the virus [25]. The proteolytic site (ALPQG) at the 2C/3A junction is highly conserved in enteroviruses and is recognized by 3C^pro^. Therefore, in this study, ALPQG was introduced based on primers designed to target both sides of the inserted BVDV E0 gene, so that the original cleavage site between 2C and 3A would not be affected after insertion of the exogenous gene.

Western blot, IFA, and sequencing all confirmed the successful rescue of the recombinant virus. Moreover, the reproductive dynamics and morphological analysis showed no influence of the exogenous gene on the replication and morphology of the recombinant virus compared to those of the parental virus. More importantly, the inserted BVDV E0 gene remained stable after 10 passages during viral passage in vitro, suggesting that the 2C/3A junction may be an ideal insertion site for exogenous genes, antigenic peptides, or markers. In addition to E0 protein, we attempted to insert the HA tag at the 2C/3A junction, which also rescued the recombinant labeled virus (data not shown). We also found that the recombinant virus could be rescued using the recognition site for 2A (LTTLG) (data not shown), suggesting that the 2C/3A junction in BEV BJ101 is suitable for insertion of an exogenous antigen, and the used hydrolysis sites for 2A/3C^pro^ will not affect the integrity of the 2C/3A protein structure or expression of exogenous genes.

The prevalence of antibodies against BEV-E was reported to be generally lower than the prevalence of antibodies against BEV-F in cattle populations [26]. Therefore, BJ101, as an EV-E virus, may be more suitable as a live virus vaccine vector than EV-F. In addition, the BEV BJ101 strain used in this study is confirmed to be safe with no report of pathogenesis to our knowledge. Nevertheless, there are two limitations of the use of BEV as a live viral vector [16]. First, the high level of pre-existing antibodies to BEV may prevent the vaccine vector from eliciting a strong immune response [27]. In this study, the mice were only infected but did not show any adverse clinical symptoms. We detected both anti-BVDV E0 and anti-BEV VP1 antibodies in BALB/c mice vaccinated with viruses. Mice vaccinated with the parental virus produced only antibodies against BEV VP1, whereas mice vaccinated with the recombinant virus rBEV-E0 produced antibodies against both VP1 and E0 with high titers. Second, like other picornaviruses, the virion has limited space but is small enough to accommodate exogenous sequences. In previous studies, the possibility of inserting only a few or dozens of amino acids (such as exogenous epitopes and exogenous markers) was explored [28], and the full-length BVDV E0 gene inserted in the present study reached up to 227 amino acids, far exceeding the length of previously inserted sequences. This attempt to insert the maximum virus protective code into the BEV viral vector can maximize the immunogenicity and offer more complete protection from disease.

## Conclusion

We successfully constructed and rescued the recombinant virus rBEV-E0, and demonstrated that insertion of the exogenous gene BVDV E0 at the 2C/3A protein junction did not affect the biological characteristics of the recombinant virus and induced an effective immune response in vivo. This recombinant virus can offer a useful tool for the simultaneous prevention and control of BEV and BVDV, as well as in further research to find appropriate insertion sites for other exogenous genes or markers. Therefore, rBEV-E0 provides an ideal technical platform for the subsequent insertion of exogenous genes, research on the mechanism of action, and further development of viral vector vaccines.

## Supplementary information

**Additional file 1.** The Optimized sequence of BVDV-E0 (Length: 681 bp)

**Additional file 2: Figure S1.** GpBlueScript(−) Vector **Figure S2** Modified GpBlueScript(−) Vector

**Additional file 3: Table S1.** The raw data of the anti-BEV VP1 antibodies detection in experimental mice **Table S2.** The raw data of the anti-BVDV E0 antibodies detection in experimental mice

## Data Availability

The data used to support the findings of this study are included in this published article.

## Abbreviations

ANOVA: Analysis of variance
BEV: Bovine enterovirus
BSA: Bovine serum
BVD: Bovine viral diarrhea
BVDV: Bovine viral diarrhea virus
CPE: Cytopathic effects
DMEM: Dulbecco’s modified Eagle’s Medium
ELISA: Enzyme-linked immunosorbent assay
FBS: Fetal bovine serum
FITC: Fluorescein isothiocyanate
HA: Hemagglutinin
HRP: Horseradish peroxidase
IFA: Immunofluorescence analysis
mAb: Monoclonal antibody
MDBK: Madin-Darby bovine kidney
PBS: Phosphate buffered saline
PBST: Phosphate buffered saline (1% Tween 20)
PcAb: Polyclonal antibody
PCR: Polymerase chain reaction
SEM: Standard error of the mean
TCID_50_: Tissue culture infective dose
TMB: 3,3′5,5′-Tetramethylbenzidin
