# Supplementary material for: Experimental immunization of mice with a recombinant bovine enterovirus vaccine expressing BVDV E0 protein elicits a long-lasting serologic response
Source: Virol J. 2020 Jul 1;17:88. doi: 10.1186/s12985-020-01338-6 (PMC7331136; doi:10.1186/s12985-020-01338-6)
Supplement: Supplementary file 1 — Additional file 1. The Optimized sequence of BVDV-E0 (Length: 681 bp) [file 12985_2020_1338_MOESM1_ESM.docx]

Additional File 1

The Optimized sequence of BVDV-E0 (Length: 681bp)

GAGAACATCACCCAGTGGAACCTGCAGGACAACGGCACCGAGGGCATCCAGAGGGCCATGTTCCAGAGGGGCGTGAACAGGAGCCTGCACGGCATCTGGCCCGAGAAGATCTGCACCGGAGTGCCCAGCCATCTGGCCACCGACACCGAGCTGAAGGCCATCCACGGCATGATGGACGCCAGCGAGAAGACCAACTACACATGCTGCAGGCTGCAGAGGCACGAGTGGAACAAGCACGGATGGTGCAACTGGTACAACATCGAGCCCTGGATCCTGCTGATGAACAAGACCCAGGCCAATCTGACCGAGGGCCAGCCCCTGAGAGAATGCGCCGTGACCTGCAGGTACGACAGGAACAGCGACCTGAACGTGGTGACCCAGGCCAGAGACAGCCCCACCCCTCTGACAGGCTGCAAGAAGGGCAAGAACTTCAGCTTCGCTGGCATCCTCGTGCAGGGCCCCTGCAATTTCGAGATTGCCGTGTCCGACGTCCTGTTCAAGGAGCAGGACTGCACCAGCGTGATCCAGGACACCGCTCACTACCTGGTGGACGGCATGACCAACACCCTGGAGTCCGCTAGGCAGGGCACAGCCAAGCTGACCACCTGGCTGGGAAAGCAGCTGAGAATCCTGGGCAAGAAGCTGGAGAACAAGTCCAAGACCTGGTTCGGCGCCTACGC
