# Supplementary figures and images for: Experimental immunization of mice with a recombinant bovine enterovirus vaccine expressing BVDV E0 protein elicits a long-lasting serologic response
Source: Virol J. 2020 Jul 1;17:88. doi: 10.1186/s12985-020-01338-6 (PMC7331136; doi:10.1186/s12985-020-01338-6)

Additional File 2


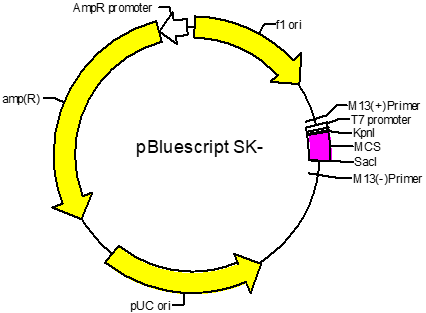

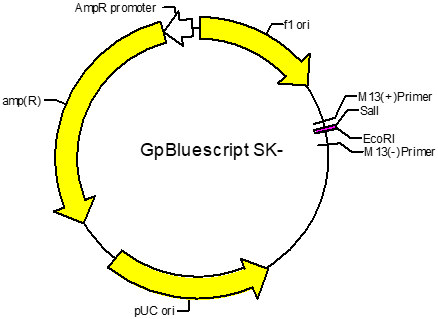


Fig.1 GpBlueScript(-) Vector Fig.2 Modified GpBlueScript(-) Vector

Supplement: Supplementary file 2 — Additional file 2: Figure S1. GpBlueScript(−) Vector Figure S2 Modified GpBlueScript(−) Vector [file 12985_2020_1338_MOESM2_ESM.docx]
